# The optimized preparation of HA/L-TiO_2_/D-TiO_2_ composite coating on porous titanium and its effect on the behavior osteoblasts

**DOI:** 10.1093/rb/rbaa013

**Published:** 2020-05-03

**Authors:** Xi Fu, Xingyu Zhou, Pin Liu, Hewei Chen, Zhanwen Xiao, Bo Yuan, Xiao Yang, Xiangdong Zhu, Kai Zhang, Xingdong Zhang

**Affiliations:** National Engineering Research Center for Biomaterials, Sichuan University, No. 29, Wangjiang Road, Chengdu 610064, China

**Keywords:** porous titanium, hydroxyapatite, titanium dioxide, composite coating, osteoblasts

## Abstract

Various surface bioactivation technology has been confirmed to improve the osteogenic ability of porous titanium (pTi) implants effectively. In this study, a three-layered composite coating, i.e. outer layer of hydroxyapatite (HA), middle layer of loose titanium dioxide (L-TiO_2_) and inner layer of dense TiO_2_ (D-TiO_2_), was fabricated on pTi by a combined processing procedure of pickling, alkali heat (AH), anodic oxidation (AO), electrochemical deposition (ED) and hydrothermal treatment (HT). After soaking in simulated body fluid for 48 h, the surface of the AHAOEDHT-treated pTi was completely covered by a homogeneous apatite layer. Using MC3T3-E1 pro-osteoblasts as cell model, the cell culture revealed that both the pTi without surface treatment and the AHAOEDHT sample could support the attachment, growth and proliferation of the cells. Compared to the pTi sample, the AHAOEDHT one induced higher expressions of osteogenesis-related genes in the cells, including alkaline phosphatase, Type I collagen, osteopontin, osteoclast inhibitor, osteocalcin and zinc finger structure transcription factor. As thus, besides the good corrosion resistance, the HA/L-TiO_2_/D-TiO_2_-coated pTi had good osteogenic activity, showing good potential in practical application for bone defect repair.

## Introduction

Titanium (Ti) and its alloys have been widely used in the repair of large bone defects at load-bearing sites due to their good biocompatibility, mechanical strength and corrosion resistance but they also face the great challenges of biological fixation, stress shielding and service life [1]. With adjustable mechanical strength and allowed tissue ingrowth, porous Ti (pTi) was paid much attention in recent years. It not only retains the advantages of dense Ti (dTi) but also reduces the disadvantage of stress shielding of Ti-based implants because of its declined elastic modulus. Thus far, various methods for preparation of pTi, including plasma spraying [2, 3], powder sintering [4], slurry foaming [5, 6], foam impregnation [7, 8], 3D printing [9, 10] and so on, were developed and contributed to the biomedical applications. Even so, the biological inertness of Ti metal in nature cannot be altered by introduction of porous structure. This biological inertness would weaken the implant’s osteointegration with host bone to some extent. When subjected to stimulation of long-term stress, the interface between the implant and the host bone was easy to become unstable, resulting in the loosening and failure of the implant [11]. Besides, for its elevated specific surface area, pTi could have increased corrosion rate as compared to dTi and thus taking additional effect on the biological performance. When contacting with body fluids, the dissolution of surface oxide film alternating with the repassivation would speed the release of metal ions from the implant and the formation of wear debris, which could cause inflammation and pain. Therefore, exploring the suitable bioactivation technology for pTi and endowing it with improved bioactivity as well as corrosion resistance are of great significance to prolong the clinical lifespan of pTi implant.

Surface modification is a good choice for improving the biological performance of a metal implant. Plasma spraying hydroxyapatite (HA) coating has been successfully used in orthopedic implantable devices and achieved satisfactory outcomes in clinic [12, 13]. However, this linear technique is difficult to produce the homogeneous surface coating in the inner pores of the porous scaffolds. Therefore, other chemical and electrochemical methods, including acid and alkali treatment, alkali heat (AH) treatment, anodic oxidation (AO), micro-arc oxidation (MAO) and so on [14–21], were developed to make the surface oxidation layer with different surface micro/nano structure on pTi, which could improve the bioactivity of the implant. Moreover, the oxidation layer chemically bonded to the Ti substrate can lead to a higher bonding force than the physically adhered coating and thus has good stability under physiological environment. Fujibayashi *et al*. [22] firstly reported the osteoinduction of pTi with special chemical and thermal treatments after implantation into the dorsal muscles of mature beagle dogs for 12 weeks. Kim *et al*. found that in the fabricated HA/TiO_2_ composite coating, the intermediate TiO_2_ layer improved the adhesive strength of HA coating and Ti substrate [23]. Based on the liquid deposition, biomimetic mineralization is another available method suitable for homogeneous apatite formation on porous scaffolds. By soaking in simulated body fluid (SBF) or other calcifying solutions, a biomimetic apatite could form on the Ti surface, which has been widely reported to promote the activity of osteoblasts as well as enhance osteointegration and osteoinduction of the implants [6, 24–26].

In recent years, various electrochemical methods were paid much attention to modify metal implants. One hand, appropriate AO treatment could not only endow the implant with special surface micro/nano structure to enhance its bioactivity but also increase its corrosion resistance [20]. On the other hand, electrochemical deposition (ED) has been confirmed to produce HA coating with controllable thickness on pTi [27, 28]. In our previous studies, a combined strategy of chemical and electrochemical treatments, i.e. AH and ED, on porous titanium was developed to fabricate an HA/TiO_2_ composite coating, whose double-layered structure endow the implant with strong adhesive strength and good bioactivity [29]. On this basis, a three-layer coating, which was composed of the inner anodized dense TiO_2_ layer (D-TiO_2_), the intermediate loose TiO_2_ gel layer (L-TiO_2_) and the outer HA layer, was prepared by an additional AO treatment. The composite coating showed the significantly improved corrosion resistance [30]. In the present study, the three-layered HA/L-TiO_2_/D-TiO_2_ coating was further optimized by adjusting the processing parameters. The biological performance of the resultant HA/L-TiO_2_/D-TiO_2_ coated pTi was evaluated by *in vitro* bioactivity tests and cell experiments to verify its prospect in practical applications.

## Materials and methods

### Material preparation

**Figure 1:**
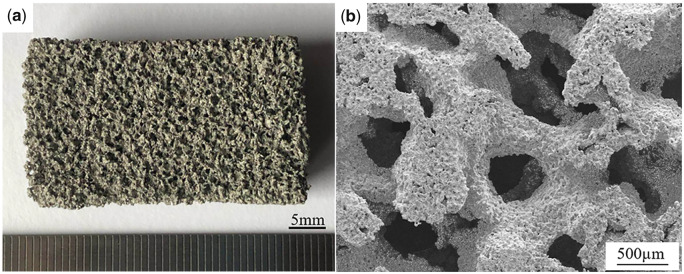
Digital photograph (**a**) and SEM image (**b**) of the pTi samples.

The pTi blocks was prepared by an improved foam impregnation method according to our previous study [8]. It had a porosity of ∼70% and average macroporous diameter of ∼360 μm, as shown in Fig. 1. The disc samples (*Φ* 9 × 2 mm^3^) were in turn cleaned with petroleum ether, acetone, ethanol absolute, and deionized water for 15 min, followed by the special chemical and electrochemical treatments. Firstly, the excess oxide layer on the surface was removed by using a mixed acid solution of 1.0 or 0.5 vol.% HF and 10 vol.% HNO_3_.T Secondly, the samples were immersed in 5 M NaOH solution at 60°C for different times and then heated to 600°C at the heating rate of 5°C/min in a muffle furnace. After holding for 1 h, the AH-treated samples were obtained by cooling with the furnace. Thirdly, the AH samples were placed in an electrolytic cell containing 0.005 M H_3_PO_4_ solution for AO treatment, where the sample and the annular graphite electrode were used as the anode and cathode, respectively. The oxidation was carried out in a constant current mode, and then the samples were placed in deionized water at 90°C for 30 min to get the AHAO samples. Finally, the pulse ED was performed on the AHAO samples at 80°C in an electrolytic cell containing electrolyte (2.5 mM Ca(NO_3_)_2_.4H_2_O, 1.5 mM NH_4_H_2_PO_4_, 0.1 M NaCl, Ca/P = 1.67) according to our previous method [30]. The material sample, ring-shaped graphite electrode and saturated calomel electrode were used as cathode, anode and reference, respectively. A pulse current (60 mA/cm^2^, 1 s on time; 0 mA/cm^2^, 15 s off time; 100 cycles) was applied to deposit HA coating on porous titanium, and then the AHAOED samples were hydrothermally treated in an autoclave at 130°C for 2 h.

### Material characterization

The surface microstructure, cross-section appearance and elemental composition of the surface-modified pTi samples were observed by a scanning electron microscope (SEM, S4800, Hitachi, Japan) equipped with an energy dispersive spectroscopy (EDS).

### The *in vitro* bioactivity evaluation

The *in vitro* bioactivity of the surface-modified pTi samples was evaluated by the classical methods, i.e. soaking in SBF based on the formula of Kokubo [31] to observe the apatite formation. After immersion in SBF at 37°C for different times (12, 24 and 48 h), the samples were removed, washed with deionized water and dried overnight at 60°C. The deposits formed on the sample surface were confirmed by SEM observation (S4800, Hitachi, Japan).

### Cell culture

Murine MC3T3-E1 pre-osteoblasts were selected as cell model to evaluate the osteogenic activity of the AHAOEDHT samples, i.e. pTi with surface three-layered HA/L-TiO_2_/D-TiO_2_ composite coating. The pTi samples without surface treatments were used as control. Before cell seeding, all the samples (Φ 9 × 2 mm^3^) were sterilized in an autoclave. The cell seeding density was 1 × 10^4^ cells/disk. The α-MEM containing 10% fetal bovine serum (GEMINI, AU) and 1% penicillin/streptomycin (Sigma, USA) was used for cell culture, which was carried out in an incubator with 5% CO_2_ at 37°C.

### Cell attachment, growth and proliferation

The cell attachment morphology on the sample surface was observed by SEM (S4800, Hitachi, Japan). After culturing for 1, 3 and 5 days, the samples were taken out, washed with phosphate buffered saline and fixed in 2.5% glutaraldehyde solution at 4°C. Next, the samples were dehydrated in series dilutions of ethanol (50–100%), dried by critical-point drying method and sprayed with gold for SEM observation.

Fluorescein (FDA, Sigma, USA) and propidium iodide (PI, Sigma, USA) staining were used to evaluate the growth of the cells on both samples. After culturing for 1, 3 and 5 days, the cells were stained with 0.1% FDA/PI solution and then observed by a laser confocal microscope (CLSM, Laica-TCS-SP5, Germany).

The cell proliferation assay was performed using a cell counting kit-8 (CCK-8, DOJINDO, Japan) method. After culturing for 1, 3 and 5 days, the medium in each well was removed and the CCK-8 solution was added. After incubating for 2 h in dark, the absorbance was measured at the wavelength of 450 nm by a microplate reader (BioTek, EON, USA).

### Osteogenic gene expressions

After the cells were cultured on both samples for 4, 7 and 14 days, the RNA was extracted from the cells with an R Neasy Mini Kit RNA (Qiagen, Germany) and then reverse-transcribed into cRNA by an iScript TM cDNA Synthesis kit (Bio-Rad, USA). A real-time reverse transcriptase polymerase chain reaction (RT-PCR) was used to detect the expression of osteogenesis-related genes including alkaline phosphatase (ALP), Type I collagen (COL-I), osteopontin (OPN), osteoclast inhibitor (OPG), osteocalcin (OC) and zinc finger structure transcription factor (OSX). Primer sequence are shown in Table 1. The relative expressions of the target genes were calculated by the ΔΔCt-value method, and GAPDH was used as an internal reference to normalize the data.

**Table 1:** Primer sequence of gene

Gene	Forward primer sequence (5’–3’)	Reverse primer sequence (5’–3’)
GAPDH	ACCCAGAAGACTGTGGATGG	CACATTGGGGGTAGGAACAC
ALP	CCAGCAGGTTTCTCTCTTGG	GGGATGGAGGAGAGAAGGTC
COL-I	GAGCGGAGAGTACTGGATCG	GTTCGGGCTGATGTACCAGT
OPN	TCTGATGAGACCGTCACTGC	AGGTCCTCATCTGTGGCATC
OPG	TCCTGGCACCTACCTAAA	CACCTGAGAAGAACCCATC
OC	GGACCATCTTTCTGCTCACTCTG	TTCACTACCTTATTGCCCTCCTG
OSX	GCTGCCTACTTACCCGTCTG	AGGTTTGCCTGCACCACTC

### ALP activity

After culturing for 4, 7 and 14 days, the cells on both materials were harvested and then lysed with RIPA Lysis Buffer (Boster, Wuhan, China). The supernatants were collected and centrifuged at 8000 rpm and 4°C for 10 min. The total proteins in the specimens were measured using a Pierce^TM^ BCA Protein Assay Kit (Thermo Scientific, USA). ALP contents in the specimens were tested by a commercialized ALP assay kit (Biyuntian, China) according to the specifications. The relative ALP per unit protein in the specimens was calculated to normalize the data.

### Statistical analysis

All quantitative data were calculated from the results of at least three parallels and expressed as mean ± standard deviation. The statistical analysis was carried out using one-way analysis of variance in a SPSS software. A *P *<* *0.05 was considered as the statistically significant difference.

## Results and discussion

### Effect of processing parameters on the surface microstructure of the pTi samples

**Figure 2:**
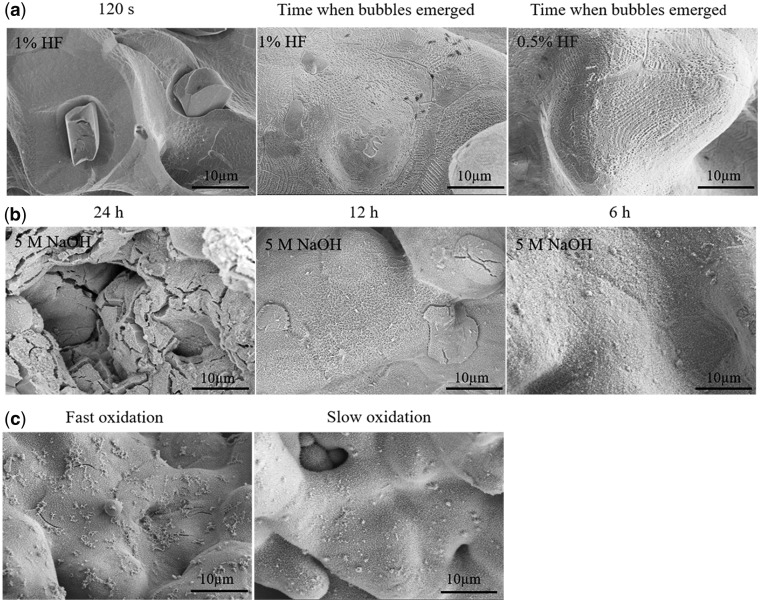
Effect of the processing parameters on the surface microstructure of the pTi samples: (**a**) different pickling conditions; (**b**) different alkali treating time; and (**c**) different anodizing rate.

Generally, a pickling procedure was needed to remove off the extra oxidation layer on pTi after machining. A mixed HF/HNO_3_ solution was used in the present study, and Fig. 2a shows the effect of pickling time and pickling concentration on the surface microstructure of the pTi sample under ultrasonic vibration condition. It could be seen that under the three treating conditions, the extra oxides on the surface could be cleared well. When using the mixture of 1.0 vol.% HF and 10.0 vol.% HNO_3_, the longer pickling time (120 s) would lead to some small protrusions occurred on the surface. If the pickling time was shorten to take the surface bubbled continuously, the protrusions disappeared but the acid etching traces could be seen obviously. If further deceased the HF concentration to 0.5 vol.% and stopped the pickling when the bubbles emerged in large numbers, the similar cleaning effect could be achieved and the surface showed the largely decreased acid etching traces. The surface protrusions could lead to the coating exfoliation easily and thus was adverse to subsequent surface modification. Therefore, a good pickling condition for pTi was acquired by using the mixture of 0.5 vol.% HF and 10 vol.% HNO_3_ and setting the time when the bubbles emerged in large numbers as the pickling time.

After pickling, an AH treatment was applied to the pTi samples. Figure 2b shows the surface microstructure of the AH-treated samples under different alkali treatment conditions. When treating the sample with 5.0 M NaOH at 60°C, the surface microstructure changed obviously with the treating time increasing from 6 to 24 h. At 24 h, lots of non-penetrating cracks appeared on the surface. When the treating time declined to 6 h, no obvious cracking could be observed. It is known that the occurrence of cracking would increase the risk of crevice corrosion. Therefore, the longtime of alkali treatment could hurt the corrosion resistance of pTi. The 6 h of alkali treatment would be the optimal condition and the resultant AH-treated samples were then used for next AO treatment.

To endow pTi with good corrosion resistance, an AO treatment was performed on the AH-treated samples in 0.005 M H_**3**_PO_4_. Under continuous electromagnetic stirring, two different oxidation rates, i.e. fast and slow oxidation, were applied. The fast oxidation meant that the samples were oxidized at 10 mA until the voltage climbed to 30 V, and the slow oxidation indicated that the samples were in turn oxidized at 1 mA for 30 min, 5 mA for 30 min and 10 mA until the voltage reached 30 V. Figure 2c shows the surface microstructure of the AHAO-treated samples. The D-TiO_2_ layer formed between the pTi substrate and the surface L-TiO_2_ layer resulted from AH treatment, as was in line with our previous report [30]. Under fast oxidation, some microcracks occurred on the surface, and the observation on the cross-section morphology showed that the L-TiO_2_ layer had the tendency to fall off. However, when slow oxidation was applied, no obvious morphological change was found on the L-TiO_2_ layer and there was also no shedding sign of the coating. It is known that during anodizing, there are not only the changes of valence state of Ti ions but also the generating oxygen. The fast oxidation would produce a large amount of oxygen within a short time, which could not in time released from the surface L-TiO_2_ layer and thus result in the breakdown of the coating. But under slow oxidation, the reactive oxygen could escape from the L-TiO_2_ layer, whose integrity was then maintained.

### Morphology and elemental composition of the composite coating on the pTi samples

**Figure 3:**
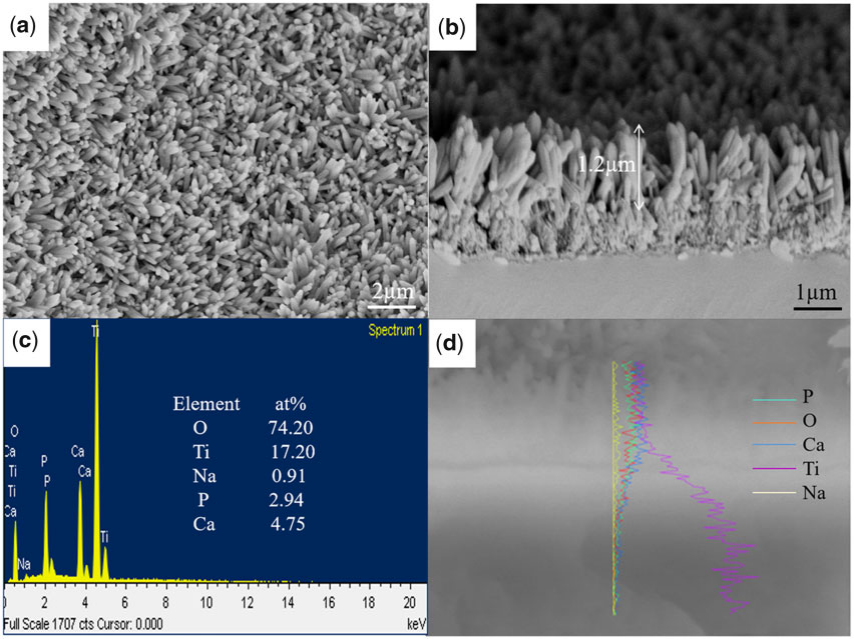
Surface morphology (**a**), cross-section morphology (**b**), surface elemental composition (**c**) and cross-section elemental composition (**d**) of the HA/L-TiO_2_/D-TiO_2_-coated pTi samples.

Based on the previously optimized electrochemical parameters [29, 30], the further ED process was performed on the AHAO-treated pTi samples. Figure 3 shows surface morphology and elemental compositions of the final products, i.e. AHAOEDHT-treated pTi. After ED and hydrothermal treatment (HT) processes, a layer of homogeneous rod-like deposits formed on the outer surface of the sample. From the cross-section image, the three-layered composite coating could be seen clearly. The outer layer had the thickness of about 1.2 μm, the intermediate layer was about 1.0 μm, and the innermost layer was dense and only about 60 nm. Born *et al*. [32] found that the thickness of the anodized film on Ti metal was positively related to the applied voltage, and the increasing rate was about 2.2 nm/V. On the basis of calculation, the thickness of anodized layer was 66 nm, as was in accordance with our experimental result. In addition, the EDS analysis confirmed that the, in turn, occurred elements from the inside to the out were Ti, O, Na, P and Ca, corresponding with the different surface treatments. Na element occurred on the middle layer confirmed the formation of titanate resulted from the AH treatment, as was in line with the previous reports [33]. The Ca/P ratio in the outer layer was 1.62 and close to the stoichiometric 1.67 of HA, indicating the HA coating.

### Apatite-forming ability

**Figure 4:**
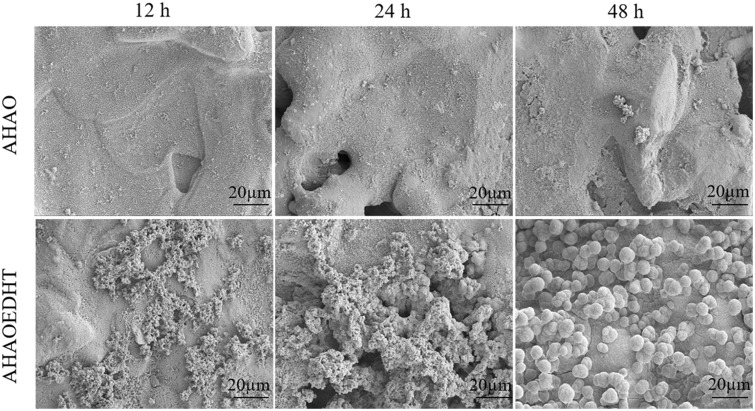
Apatite formation on the AHAO and AHAOEDHT samples after soaking in SBF for 12, 24 and 48 h.

Figure 4 shows the typical SEM images of both AHAO and AHAOEDHT samples after soaking in SBF for 12, 24 and 48 h. For AHAO sample, there was almost no deposits formed at 12 and 24 h, and a few amount of substances could be found in the local areas of the sample surface at 48 h. However, for AHAOEDHT sample, the half surface was covered by a layer of deposits at 12 h. At 24 h, both thickness and covering area of the deposits further increased. At 48 h, the apatite deposits covered the sample surface completely. The results proved that the AHAOEDHT sample had higher bioactivity than the AHAO one, as the surface HA coating on the former endowed it with the enhanced apatite-forming ability. Thus far, SBF immersion has been widely accepted for evaluating the bioactivity of a biomaterial [34]. Although the previous studies confirmed that pTi with specific surface modification including AH treatment could increase the material’s bioactivity, it often required different time to form apatite after soaking in SBF [35–37]. Besides, the AH or other surface-modified pTi was reported to have osteoinductivity when ectopically implanted into the body of the experimental animal for 5 months or longer time [14, 38, 39]. With additional AO treatment, both AHAO and AHAOEDHT samples exhibited the increased corrosion resistance in our previous study [30]. However, the AHAO sample showed weaker apatite-forming ability than the AHAOEDHT one. According to the theory of biomaterials’ osteoinductivity, the apatite formation on the biomaterial surface under physiological environment is one of the essential conditions [40]. Therefore, the rapid apatite-forming ability meant that the AHAOEDHT-treated pTi could have high biological performance.

### Effect of the HA/L-TiO_2_/D-TiO_2_ composite coating on the behavior of osteoblasts

**Figure 5:**
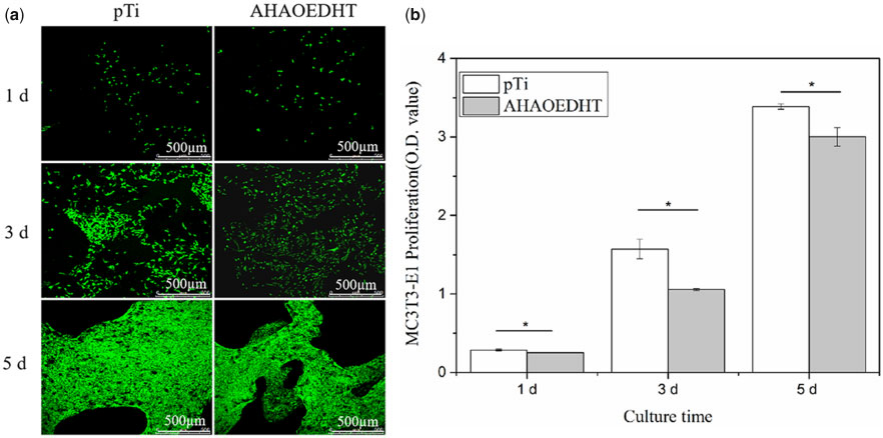
The CLSM observation on the morphologies (**a**) and CCK-8 analysis for the proliferation (**b**) of MC3T3-E1 cells cultured on the pTi and AHAOEDHT samples. *P < 0.05 Significant as compared to pTi group.

**Figure 6:**
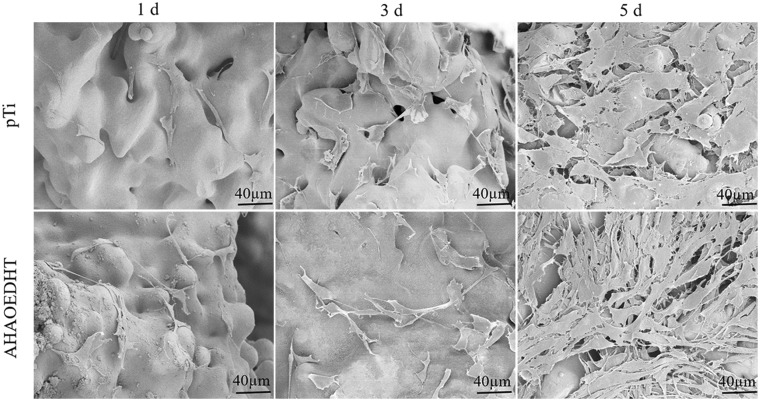
The typical SEM images of MC3T3-E1 cells cultured on the pTi and AHAOEDHT samples for 1, 3 and 5 days.

Figure 5a shows the CLSM images of MC3T3-E1 pre-osteoblasts cultured on both pTi samples for 1, 3 and 5 days. It could be seen that there were a large amount of living cells stained with green on the surface of both samples, and almost no dead cells stained with red were found. With time prolongation, more and more living cells occurred on the surface and had the tendency to migrate into the inner pores of the scaffolds. Figure 5b shows the CCK-8 assay for the proliferation of MC3T3-E1 cells cultured on both pTi samples for 1, 3 and 5 days. In line with the CLSM observation, the number of the living cells increased with the increasing of the culturing time on both groups. However, the quicker cell proliferation was found in pTi than AHAPEDHT group at each time point. Figure 6 shows the SEM images of MC3T3-E1 cells attached on the surface of both pTi samples. It further confirmed the above results of the CLSM observation and CCK-8 assay. With time prolongation, more and more cells attached on the sample surface and had the stretched pseudopods, presenting a good spreading state. At 5 days, the cells took on a congregated morphology on both groups. Based on the magnification images at 3 days, it could be found that there was a little difference in cell spreading morphology between the pTi and AHAOEDHT groups. The cells on the pTi sample took on a relatively round morphology with shorter pseudopods. But the rough AHAOEDHT sample made the cells flattened, whose pseudopods were less and longer.

**Figure 7:**
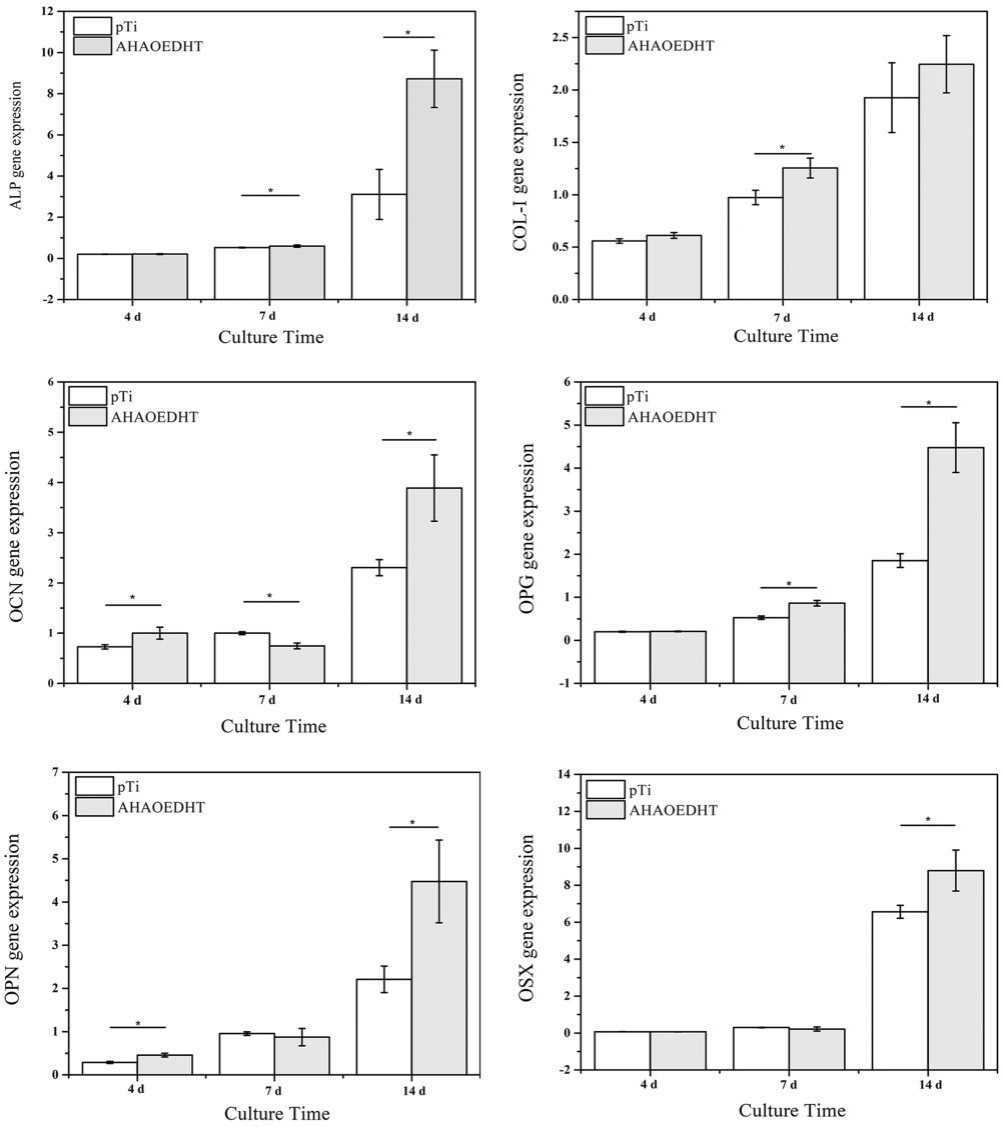
PCR Analysis for the expressions of osteogenesis-related genes in MC3T3-E1 cells cultured on the pTi and AHAOEDHT samples for 4, 7 and 14 days. *P < 0.05 Significant as compared to pTi group.

**Figure 8:**
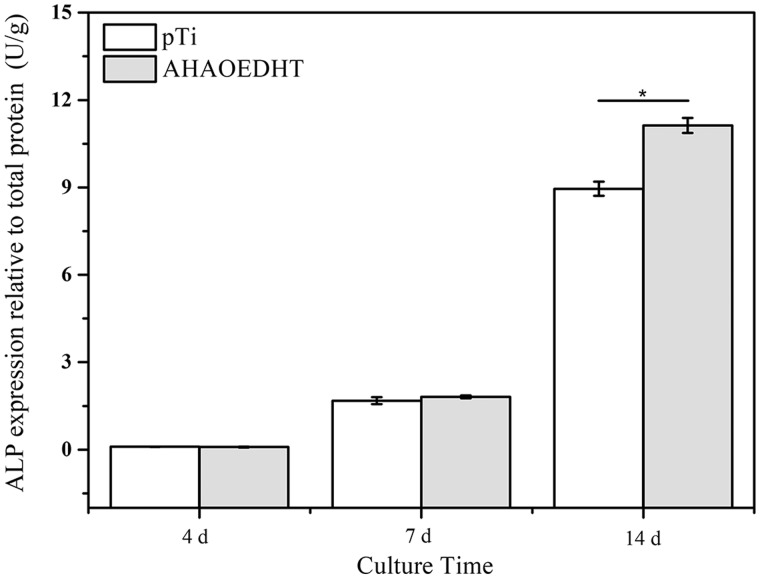
The ALP activity in MC3T3-E1 cells cultured on the pTi and AHAOEDHT samples for 4, 7 and 14 days. *P < 0.05 Significant as compared to pTi group.

Figure 7 shows the expressions of osteogenesis-related genes, including COL-I, ALP, OPN, OPG, OCN, OSX, in MC3T3-E1 cells cultured on both pTi samples for 4, 7 and 14 days. In general, all the genes had the increased expressions with the increasing of culturing time in both groups. However, the HA/L-TiO_2_/D-TiO_2_ composite coating influenced the expressions of osteogenesis-related genes in the cells significantly. For the early-stage markers of osteoblastic differentiation, i.e. COL-I and ALP, there was no significant difference in their expressions between both groups at 4 days. But the AHAOEDHT group showed much higher COL-I expression at 7 days and ALP expression at 14 days than the pTi one. For the middle and late stage markers, including OPN, OSX, OPG and OCN, the AHAOEDHT group showed much higher expressions of them than the pTi one when the culturing time prolonged to 14 days. Figure 8 shows the ALP activity of MC3T3-E1 cells cultured on both pTi samples for 4, 7 and 14 days. The similar tendency with ALP gene expression was found. With time prolongation, the ALP activity increased obviously in both groups. At 4 and 7 days, there was no obvious difference in ALP activity between both groups. But at 14 days, the AHAOEDHT group showed higher ALP activity than the pTi one.

It is known that surface chemical composition and micro/nano structure play important role in regulating the response of the cells. Many previous studies confirmed that either chemical or electrochemical treatments would change the surface composition and structure of Ti-based implants, and thus mediate the behavior of osteoblasts or mesenchymal stem cells (MSCs) [26, 41–44]. In current study, the pTi sample without any surface treatment had a relatively smooth surface, which was favorable for the attachment and proliferation of MC3T3-E1 pre-osteoblasts. The AHAOEDHT treatment led to the formation of HA/L-TiO_2_/D-TiO_2_ composite coating on the pTi surface and thus significantly increased its surface roughness, which could affect the cell proliferation to certain extent. Liu *et al*. [45] found that sandblast combined with acid etching or HT increased the surface roughness and hydrophilicity of the only polished Ti, and the proliferation of MC3T3-E1 cells on the polished Ti was slightly higher than that on the surface-treated one.

But even so, the HA/L-TiO_2_/D-TiO_2_ composite coating could up-regulated the expressions of osteogenesis-related genes in the cells significantly and enhance the osteogenic activity of the pTi implant. Thus far, the positive role of HA coating in regulating the osteogenic activity of various osteoblasts have been widely reported [46]. Pang *et al*. [47] fabricated two types of HA coating, i.e. rod-like and flake-like micro-flower arrays, on pure Ti plates by a hydrothermal-electrodeposition method, and the cell experiments showed that compared to the sample without HA coating, both HA coated samples could promote the expressions of osteogenesis-related genes in MC3T3-E1 cells, and the rod-like HA array exhibited better effect than the micro-flower one. Du *et al*. [48] investigated the biological performance of HA crystals formed on MAO-treated Ti surface by a microwave hydrothermal (MH) method, and found that compared to the MAO-treated Ti surface, the outer HA columnar crystals resulted from MH treatment induced higher expressions of various genes including ALP, OPN, BSP and BMP-2 in MC3T3-E1 cells. Besides HA coating, surface microporous TiO_2_ layer has been confirmed to have notable promoting effect on the osteoconduction and osteoinduction of porous Ti implants [14, 33, 39, 49]. But compared to TiO_2_ coating, HA coating on Ti surface seemed have higher osteogenic activity [50]. The current study fabricated the three-layered composite coating, which combined the advantages of bioactive TiO_2_ layer and HA coating and could have better potential in inducing bone regeneration. Moreover, its good corrosion resistance evidenced by our previous study could decrease the risk of biosafety effectively in practical application of pTi implants [30]. Therefore, in the next work, the *in vivo* evaluation of the HA/L-TiO_2_/D-TiO_2_-coated pTi implants on bone defect repair will be done systematically to verify its application prospect in clinic.

## Conclusion

The present study found out an optimized pickling, AH and AO treating conditions for fabricating the HA coating via an ED technique, and thus obtained the HA/L-TiO_2_/D-TiO_2_ composite coating on the pTi surface. Firstly, using the mixture of 0.5% HF and 10% HNO_3_ for pickling of pTi, the extra oxides could be cleared completely and no protrusions occurred on the surface when the bubbles emerged in large numbers. Secondly, the AH treatment by 5 M NaOH for 6 h, a microporous L-TiO_2_ layer without any cracks was formed on the substrate. Thirdly, by adopting a slow anodizing (1 mA for 30 min, 5 mA for 30 min and 10 mA until the voltage reach to 30 V), a D-TiO_2_ layer formed between the substrate and L-TiO_2_. Finally, by an ED technique, a three-layered HA/L-TiO_2_/D-TiO_2_ composite coating formed on the pTi surface successfully. The obtained composite coating showed rapid apatite-forming ability when soaking in SBF, indicating its good bioactivity. The cell culture confirmed that compared to the pTi without surface treatment, the HA/L-TiO_2_/D-TiO_2_ coated one could significantly up-regulate the expressions of osteogenesis-related genes in MC3T3-E1 cells, presenting the enhanced osteogenic activity. In conclusion, the three-layered HA/L-TiO_2_/D-TiO_2_ composite coating could not only increase the corrosion resistance of pTi but also improve the osteogenic activity significantly and thus exhibit good prospect in practical application.

## Funding

This work was financially supported by the National Natural Science Foundation of China (81671825) and the Sichuan Science and Technology Innovation Team of China (2019JDTD0008).

*Conflict of interest statement*. None declared.
